# Employers and Employed in America

**Published:** 1905-01-21

**Authors:** 


### Modern Sociology.

### EMPLOYERS AND EMPLOYED IN AMERICA.

Paternal government is by no means popular in America, and the relations of master and man are often simply a claim to good work on the one side and good wages on the other. But of late years, and especially in those cases where a new industry has been set up in a more or less uninhabited part of the country, employers have felt bound to make provision for the housing and for the moral, social, and intellectual opportunities which their workmen need, and feeling this, they have responded with true American generosity. The style of houses provided varies with the locality, but all seem to be well-planned, and to give as much accommodation as an average family requires. If the rents seem to the English mind to be higher than a working-man could pay, it must be remembered that American wages are higher also. To the English eye, also, the houses may seem flimsy structures, being constructed of wood, but this again is a point on which the two nations differ.

Mining companies everywhere have to make housing provision for their employés, since the discovery of minerals is often the first thing to bring people into a hitherto desolate neighbourhood. But not many go so far in their provision as the Colorado Fuel and Iron Company. The houses provided by this company are for the most part unpretending structures, containing from four to six rooms; but they are an immense improvement on the old stone or mud huts which the miners used to occupy, and are always well-planned and sanitary. In some of the mining villages, or camps, as they are still called, the company keeps a model cottage, simply but tastefully furnished, not for occupation, but as an object lesson to the people, showing them what can be done without a great expenditure of money, if a little taste be employed. For the higher class of employés larger houses are provided, but for all the rent is calculated at an unvarying rate of two dollars per room per month. If all the employés were American, it might not be necessary to maintain model cottages as object-lessons, but they include, according to the report of the Labour Bureau at Washington, "32 nationalities speaking 27 languages," and many of them are drawn from "the lower classes of foreign immigrants, Italians, Austrians, Germans, and Mexicans predominating," who have primitive ideas of living and are ignorant of the laws of health. These different nationalities have to be reckoned with also in the weekly magazine published by the company, which is itself a valuable means of keeping in touch with each other the various camps, scattered over an expanse of a thousand miles, which the company owns. Some of the contents of this are printed in Italian, German, and other languages spoken by the foreigners. As, however, the children of these are to be American citizens, good schools are provided, and no fault is found if the kindergarten department of these is used practically as a day nursery. At Pueblo there is an industrial school, where crippled employés and the widows and orphans of those who have lost their lives in the company's service are taught various trades. In the same town is the central hospital of the company, while branch hospitals are established at all the camps. The number of cases treated in the hospitals during the year ending June 1903 was 82,821. In all the camps there are club-houses for the employés, and the company, wisely comprehending the manner of men with whom they have to deal, allow the sale of intoxicants of good quality at moderate prices, while they deal sternly with excessive indulgence. A wise restriction is the forbidding of "treating." One rule runs thus:—"In order to promote the temperate use of wine, beer, and liquors which may be sold in the club-house, no member or visitor shall be permitted to purchase or pay for a drink or drinks for any other member or visitor." If that rule could only be applied in this country a good deal of our drunkenness would disappear.

Clubs are a regular feature of nearly all the towns and villages which owe their existence to the interest which employers feel in their employés, but as a rule no intoxicants are sold in them. None of these, however, occupy such isolated positions as the camp of the Colorado Company. The Waltham Watch Company, which employs a great many girls, takes special concern as to the lodging and feeding of these. It has provided a well-built, sanitary, steam-heated house with 67 bedrooms, each of which two girls are expected to share. Board is provided on a generous scale. A visitor who stayed there for a week gives the menu, which he admits would seem excessive to anyone who had not sat down to table with an army of Yankee girls. Breakfast included beefsteak with baked potatoes, eggs, white and brown bread, rolls and doughnuts, butter and condiments, tea and coffee. The reporter notes the clean napkins that lay at each plate. Tea and coffee as well as pitchers of milk appeared again at dinner, in addition to soup, scalloped oysters, roast beef and mutton, celery and pickles, pudding and pie. Supper consisted of cold meat, cheese, various kinds of bread, and again tea, coffee, and milk. Two large sitting-rooms, tastefully furnished, are open to the occupants of the house at all times, and there are no vexatious restrictions as to going out and coming in, the company trusting, with good reason, to the conscience and self-respect of their workers. When it is mentioned that the girls enjoy all this for three dollars (12s. 6d.) a week, it will be seen that many of the women workers of London have cause to envy the Waltham girl watchmakers. Of the married employés many own their pretty homes, and the company is willing to lend financial assistance to any who desire to do so, although they have now ceased to build houses for these.

The food question is kept in view also by the Plymouth Cordage Company of Plymouth, Massachusetts, but it is for their male employés that these specially cater. Finding that men who could not go home to their midday meal asked for a place where they could get coffee and light refreshments, one of the directors built a dining-hall at his own expense, where a substantial meal—meat and two vegetables—could be had for 10 cents. (5d.), and pudding, tea, or coffee for 1d. more. At this price elaborate service cannot be given; the men get their dinners at the counter and themselves carry them to the tables, but the dinners are good as well as cheap This company's works are situated near the sea, and the company have constructed a safe and convenient bathing beach, with the necessary dressing-rooms for their employés, and otherwise encourage them in so many ways that one does not wonder that "Labour Day" is here a festival and not a sign of discontent. The houses for the different grades of employés differ in elaboration in proportion to the incomes of those who inhabit them, but even the cheapest of them is commodious and well-planned. A seven-roomed house with a good garden can be had for 10s. a week, and others with less bedroom accommodation for 6s. or 7s. The houses belonging to the Maryland Steel Company rejoice in a summer kitchen, built farther from the living rooms of the house than that for winter use. In other places other modifications, suggested by climate and custom are to be found, but in all there is a thoughtfulness for the well-being of those who are to occupy them which show that when the American employer takes up the notion of caring for his employés' comfort, he carries it out with true American thoroughness.

